# Mr. Chandler's Case of Laceration of the Bladder

**Published:** 1806-02

**Authors:** W. Chandler

**Affiliations:** Burgate-Street, Canterbury


### *To the Editors of the Medical and Physical Journal.*

Gentlemen,

A Man was admitted into the Kent and Canterbury Hospital with a violent contusion upon his loins, occasioned by a fall from the roof of a coach during a state of ebriety; the consequent ecchymosis extended entirely over the lumbar region and nates; he complained of most acute pain in the lower part of the abdomen, and ejected from the stomach, frequently, large quantities of dark green fluid; the vomiting was increased by any fluids taken into the stomach; his pulse was quick and weak; the skin hot and dry, accompanied with great thirst; his countenance particularly expressive of fear and despair. Not having evacuated the bladder for some time previous to to the accident, nor since his admission, which was eight hours after the fall, a catheter was introduced, by which means a small quantity of healthy urine was discharged, but the last drops which escaped upon withdrawing the catheter were of a dark bloody hue. From this he appeared somewhat relieved, but all his sufferings very soon returned; twelve ounces of blood were taken from the arm, saline effervescing draughts, and fomentations to the loins and abdomen were ordered; the warm bath was used, and cathartic enemas were administered. The catheter was again introduced, and half a pint of bloody urine removed, which did not relieve him as in the morning. An elastic gum catheter was allowed to remain in the bladder, that the urine might flow off as fast as it was secreted; the retention of it, in the bladder, appeared to increase his uneasiness considerably, and therefore it was withdrawn early in the morning.

On the following day, his symptoms were increased, he was evidently weaker; the abdomen was now much swollen, and extremely painful to the touch; he could lie only on one side; any thing taken by the mouth occasioned vomiting. He expressed a wish to repeat the warm bath, having found himself on the first day easier when wholly immersed in it; the glyster was also repeated, and he took two grains of opium every four hours. He had some rigors in the day; in the evening I found him very low, delirious, but in less pain; some brandy and water, for which he asked, remained upon his stomach, and he took the saline draught again at bed time; nutritive broth glysters were injected; a catheter was again passed, but no water discharged; about two o'clock in morning he died. Upon inspecting the body, the intestines in some parts were highly inflamed, the peritoneum in the hypogastric region, from the umbilicus to the spine and around the pelvis, was of a dark livid hue; the bladder, which was collapsed, was found ruptured at the fundus, and the aperture would readily admit four or five fingers; a vast quantity of bloody fluid was effused into the cavity of the abdomen; the other viscera appeared perfectly healthy; the stomach and gall bladder were contracted, the latter was nearly empty.

Since the above case occurred, Mr. Irwin, Surgeon to the 4th Dragoons (Royal Irish) had the goodness to communicate to me a similar case of laceration of the bladder, which came under his care when in Ireland.

A soldier,

A soldier, intending to rest himself, placed the handle of a large wooden mallet, with which he had been working, on the ground; then seated himself abruptly on the opposite end; this suddenly slid down, and the handle was forced through the rectum into the bladder; no urine whatever was voided by the urethra, but passed freely through the wound; and in consequence of extreme irritation, he died on the second day after the accident.

Upon examining the body, the rectum and inferior parts of the bladder were found lacerated, and a small quantity of urine had escaped into the abdomen, where it had occasioned much inflammation. It may not be improper to remark, that the subject of the first case was, the day he met with the accident, discharged from the Naval Hospital at Deal, having partial paralysis of the lower extremities.

Mr. T. S. aged twenty-seven, of spare habit, has been the subject of hernia (entro epiplocele) for five years; the intestine he could at all times return with ease, but the omentum was irreducible. On the evening of the 7th of March, after a little exertion, the truss being improperly applied, the intestine came down; he that day paid but little attention to it, in the hope that he should, as usual, be able to return it when in bed; but this time his endeavours were unsuccessful. In the afternoon of the following day, he suffered considerable pain in the tumour, accompanied by great sickness and vomiting; he then requested my father to see him, who immediately bled him, and endeavoured to return the rupture, but without effect. Aperient enemas were ordered, he took calomel and cathartic extract; a small quantity of fæces, evidently only such as had been lodged in the rectum, were discharged; the vomiting of dark bilious matter continued, attended with great pain and most distressing sensation of stricture in the direction of the diaphragm. Large doses of digitalis were given, and opiates combined with purgative clysters, which procured some ease; the taxis was continued for some time without success; he was put into the warm bath, where he remained till syncope was produced, at which time the efforts for reduction were again employed, and continued three-quarters of an hour; as no success attended these means, cold applications were next had recourse to, but without effect; and the vomiting continued.

It was now conceived that the tobacco injection, or the operation, were the only remaining means on which any reliance reliance could be placed; and whilst the preparations were making for the purpose, it occured to me, to try the tinct. ferri muriat, which has been recommended by Mr. Cline in cases of spasmodic strictures; I gave him thirty drops every quarter of an hour in barley-water; this appeared to relieve the sickness, in some degree; I again tried pressure, and in about thirty-five minutes from the first exhibition of the tincture, the intestine returned. I feel at a loss to explain the modus operandi of this medicine; its good effects, in cases of spasmodic strictures of the urethra, have indisputably placed it above all other remedies which have been employed for their removal; shall we say, that it operates in a similar manner upon hernia when strangulated? Can a spasmodic affection of the muscles of the abdomen produce such a strangulation? And are we to consider this remedy administered in full doses as an antispasmodic?

All medicines capable of producing nausea, have been considered as producing antispasmodic effects; and, in this way, it has been attempted to explain the operation of large and frequent doses of tinctura ferri muriat in spasmodic strictures.

Without intending to controvert the general position, I shall only notice that the dose recommended by Mr. Cline, is only ten drops every two minutes; that in the preceding case, I administered thirty drops every fifteen minutes; that the sickness was alleviated by it. Now, if we suppose the former dose to act by producing nausea, we shall be at a loss to explain, how it came to pass, that the latter dose checked sickness. Thus I find myself under the mortifying necessity of remaining content with a knowledge of the good to be derived from this medicine, without being able, sastisfactorily, to account for the way in which it acts. My motive for using the tinct. ferri muriat, was to preclude the necessity of having recourse to tobacco injections, which always produce such a train of unpleasant symptoms and horrid sensations to the patient, that it becomes the duty of every medical practitioner to endeavour, if possible, to supersede its use.

I shall now only add, that two days after the reduction of the intestine, the previously irreducible portion of omentum receded perfectly whilst the patient was in bed, without any efforts having been made to accomplish it. This shews it was not adherent, and that its bulk and form, form only had prevented its return; these were diminished by the relaxation and emaciation, consequent on his confinement.

I am, &c.

*Burgate-Street, Canterbury,* W. CHANDLER, Jun.

*October* 15, 1805.

